# RibAlign: a software tool and database for eubacterial phylogeny based on concatenated ribosomal protein subunits

**DOI:** 10.1186/1471-2105-7-66

**Published:** 2006-02-13

**Authors:** Hanno Teeling, Frank Oliver Gloeckner

**Affiliations:** 1 Microbial Genomics Group, Max Planck Institute for Marine Microbiology, D-28359 Bremen, Germany; 2 International University Bremen, D-28759 Bremen, Germany

## Abstract

**Background:**

Until today, analysis of 16S ribosomal RNA (rRNA) sequences has been the de-facto gold standard for the assessment of phylogenetic relationships among prokaryotes. However, the branching order of the individual phlya is not well-resolved in 16S rRNA-based trees. In search of an improvement, new phylogenetic methods have been developed alongside with the growing availability of complete genome sequences. Unfortunately, only a few genes in prokaryotic genomes qualify as universal phylogenetic markers and almost all of them have a lower information content than the 16S rRNA gene. Therefore, emphasis has been placed on methods that are based on multiple genes or even entire genomes. The concatenation of ribosomal protein sequences is one method which has been ascribed an improved resolution. Since there is neither a comprehensive database for ribosomal protein sequences nor a tool that assists in sequence retrieval and generation of respective input files for phylogenetic reconstruction programs, RibAlign has been developed to fill this gap.

**Results:**

RibAlign serves two purposes: First, it provides a fast and scalable database that has been specifically adapted to eubacterial ribosomal protein sequences and second, it provides sophisticated import and export capabilities. This includes semi-automatic extraction of ribosomal protein sequences from whole-genome GenBank and FASTA files as well as exporting aligned, concatenated and filtered sequence files that can directly be used in conjunction with the PHYLIP and MrBayes phylogenetic reconstruction programs.

**Conclusion:**

Up to now, phylogeny based on concatenated ribosomal protein sequences is hampered by the limited set of sequenced genomes and high computational requirements. However, hundreds of full and draft genome sequencing projects are on the way, and advances in cluster-computing and algorithms make phylogenetic reconstructions feasible even with large alignments of concatenated marker genes. RibAlign is a first step in this direction and may be particularly interesting to scientists involved in whole genome sequencing of representatives of new or sparsely studied eubacterial phyla. RibAlign is available at

## Background

Analysis of 16S ribosomal rRNA (rRNA) sequences is currently the de-facto gold standard for the assessment of phylogenetic relationships among prokaryotes. There are various reasons that have made the 16S rRNA gene the first choice as a phylogenetic marker, such as the presence of positions with different evolutionary rates, its universal occurrence within prokaryotes, its reasonable information content, a length that was suitable for complete sequencing when the technique started, knowledge about its secondary structure that helps with alignments and finally the presence of a comprehensive database of more than hundred thousand sequences [1]. With ARB [2], there is also a well-curated 16S rRNA database with a curated alignment and a program suite for phylogenetic reconstructions available that has gained broad acceptance among scientists worldwide.

Despite this success, trees based on 16S rRNA sequences lack resolution when it comes to elucidating the branching order of individual phyla [3]. This limits our understanding of early evolutionary splits within the prokaryotes and the degree of relatedness among individual phyla, of which some have been proposed to build super-clusters [4,5]. These issues still are matters of sometimes heated debates [6,7]. It is possible that particularly early evolution can never be fully determined because an early evolutionary boundary limits the attainable resolution. The cause for this boundary might be either (a) methodological and caused by the limited information content (i.e. mutational saturation) of single marker genes, or (b) fundamental and caused by extensive lateral gene transfer (LGT) among early prokaryotes [8-10].

Before the genomic revolution, it had been anticipated that the wealth of information from entire genomes would lead to a refined view on the tree of life. Consequently, the ever-growing availability of complete genome sequences has propelled the development of new phylogenetic methods. Some of these methods exploit information from entire genomes whereas others use only a subset. Examples are super-tree approaches that combine individual trees [11,12], methods based on comparisons of genes between organisms (shared gene content [13-16], shared gene order [15], similarities of protein folds and domains [17,18]), methods based on intrinsic DNA-signatures (e.g. skewed oligonucleotide distributions) [19] and concatenations of marker genes [4,20-27].

It is one of the big disillusions of the post-genomic era, that most of these methods fail to provide an advantage in resolution over 16S rRNA-based trees [5]. Instead, comparative genomics revealed an extent of LGT that seriously questions the applicability of the eukaryotic species concept to the world of the prokaryotes. As a result, today the tree of life must be regarded as a complex network of vertical and horizontal inheritance. The extent to which tree reconstruction is affected by LGT is still a matter of debate [28]. It has been argued that a subset of the genes, including those encoding (most) ribosomal proteins, are less likely to undergo LGT and that for these core genes a phylogeny can be reliably inferred [28-31]. Whether such a stable genetic core really exists is hard to prove and hence discussed controversially [8,11,12]. Its existence is supported by the fact that phylogenetic analysis of alleged core genes in general support the 16S-derived three domain concept and mostly also correlate with 16S rRNA analysis in detail – a congruence that is notably absent form most non-core genes [30]. From the core genes, ribosomal proteins are of particular interest because their tight interactions with the 16S and other rRNAs suggests co-evolution of these molecules. Moreover, concatenation of ribosomal protein sequences is one of the few methods that has been ascribed an enhanced resolution [5]. This is also reflected in a variety of publications on phylogenetic reconstructions that are based on this method [4,20-25,27].

As of this writing (May 2005), 224 completely sequenced eubacterial genomes are available to the public. Hence, the data set available for comparison of ribosomal protein sequences is sparse when compared to the vast amount of available 16S rRNA sequences. On the other hand, most of the known phyla have been covered by at least one sequenced representative, and the gaps are being filled quickly. In addition, most draft genome sequences contain most if not all of the ribosomal proteins, so that the method is not necessarily restricted to fully closed genomes.

## Implementation

RibAlign has been implemented in a fully object-oriented manner with REALbasic [32] and uses the high-performance Valentina object-relational database engine [33] to store sequences and related information.

New sequences can be imported from whole-genome GenBank or FASTA files. An automated screening of the annotated gene descriptions and gene names assists in the extraction of the ribosomal proteins before writing them to the database. The database and importer has currently been designed for the extraction and storage of sequences from *Eubacteria*, but future releases of RibAlign might include archaeal ribosomal protein sequences as well.

RibAlign can not only export sequences to plain FASTA format, but also has a complete built-in pipeline for generating processed input files for the PHYLIP [34] and MrBayes [35,36] phylogenetic reconstruction programs. This pipeline comprises exporting dedicated multi-headed FASTA files for a selectable subset of ribosomal proteins, alignment of the exported sequences independently for each gene, concatenation of the individual alignments into a single alignment, filtering of the less-conserved positions according to an adjustable threshold and finally conversion to PHYLIP or NEXUS format.

RibAlign does not implement its own alignment algorithm but instead uses the MAFFT program [37], which can generate high-quality alignments with good speed even when used with larger sets of sequences. MAFFT is not part of RibAlign's distribution and thus has to be obtained and installed separately [38].

RibAlign comprises a searchable, tutorial-like online help that provides detailed information on all of the program's features.

We expect the implementation of RibAlign and the underlying database to perform nicely with the upcoming flood of genome sequences, since it has been tested with 10,000 artificial entries. The current release of RibAlign requires Mac OS X and as of this writing, no decisions on possible ports to other platforms have been made. Contributions concerning this matter are welcome.

RibAlign is freely available for academic applications and can be downloaded from its website [39], which also provides screenshots of RibAlign's user interface.

## Results and discussion

### Construction and quality of the RibAlign data set

RibAlign is bundled with an example database (RibAlignDB) that contains the ribosomal protein sequences of 184 of the publicly available complete eubacterial genome sequences. This data set has been generated by importing the respective GenBank files, followed by some manual curation. The latter comprised shifting N-termini of sequences, deleting false paralogs, cross-checking of dubious annotations by InterPro [40] searches and in some cases re-annotation of falsely annotated ribosomal proteins. Despite these efforts, RibAlign's data set can by no means be regarded as well-curated. Like all genome annotations, it does contain errors. Thus, data should be checked carefully prior to using it for phylogenetic reconstructions. *Archaea *are currently not included, since (a) so far only 22 complete genome sequences of *Archaea *are publicly available (b) the monophyletic nature of the *Archaea *is under discussion [41] and most important (c) joint data set of eubacterial and archaeal sequences produce less reliable alignments due to differences in ribosome composition between both domains [42].

In summary, RibAlignDB provides a good starting point for scientists who are interested in phylogenetic reconstructions based on concatenated ribosomal protein sequence. Sequences from future genome sequences can be added relatively easy due to RibAlign's powerful import filters.

### Computational requirements

Phylogenetic reconstructions based on large alignments are very hardware-demanding, especially when likelihood-based methods are used in conjunction with resampling techniques. Even with the few available genomes today, concatenated alignments of ribosomal proteins sequences can easily exceed one million individual positions. Therefore, a selection in species and sequences has to be made for the more CPU-intensive treeing methods.

The MrBayes^1 ^phylogenetic reconstruction program is fast since it is optimized for speed. However, this speed comes at the price of high memory requirements. As an example, a tree for 120 species and 5182 amino acid positions was calculated within a few days on a dual 3.0 GHz Xeon machine, but the calculation required 8 GB of main memory even when only two chains were used (tree not shown). Thus, larger data sets require either more memory or an MPI-aware cluster running the MPI-version of MrBayes.

**Figure 1:**
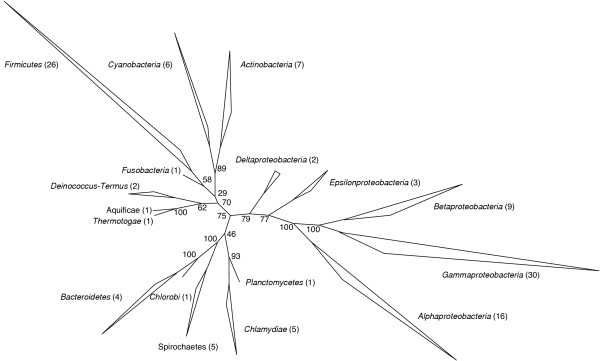
Unrooted maximum-likelihood tree based on concatenated ribosomal proteins for 120 eubacterial species. The following 37 sequences were concatenated: RplABCDFIJKLMNOPQRSTUVW, RpmA, RpsBCDEFGHIKLMOQRST, filter by a 40% positional conservation filter resulting in 5182 amino acid positions. The tree was calculated from this alignment using the ProML program of the PLYLIPackage (settings: best tree search mode; JTT model of amino acid substitution; one category of sites with a constant evolutionary rate; no weights; rough-type of analysis with global rearrangements). Bootstrapping was carried out with 100 replicates. Numbers in parentheses refer to the numbers of species that make up the respective branch.

The PHYLIP package^2 ^is comprised of programs for different kinds of phylogenetic analysis. For a bootstrapped maximum-likelihood tree with ProML, raw computing power is the limiting factor. As an example (Figure 1), a ProML run with 100 replicates for the above-mentioned data set (120 species with 5182 amino-acid positions each) took more than three months to compute on a ten-node cluster of dual 2.8 GHz Xeon processors (with each node calculating ten trees). Future improvements in processor speed and the growing use of cluster computing in bioinformatics will hopefully keep up with the increasing computational demand. However, since the computational requirements increase progressively with alignment size and the number of species, a situation like with today's 16S rRNA phylogeny is likely, where a de novo tree based on all available sequences cannot be computed any more.

### Tree topology

In the above-mentioned maximum likelihood tree calculated from concatenated ribosomal protein subunit sequences, all major phyla are well resolved (Figure 1). The topology is in good agreement with the widely accepted 16S rRNA-derived topology and also with a recently published tree based on concatenated ribosomal proteins subunit sequences [23].

The corresponding MrBayes tree showed the same topology (data not shown). Posterior probabilities computed from 13,000 trees showed good support for several of the earlier proposed super-clades, namely affiliation of *Actinobacteria *and *Cyanobacteria *[4], of *Chlamydiae *and *Planctomycetes *[20], and of *Chlorobi *and *Bacteroidetes *[43]. However, good statistical node support does not preclude tree reconstruction artifacts [44]. For example, different evolutionary rates might lead to artificial clustering of fast-evolving species due to long branch attraction. In addition, a common thermophilic lifestyle like that of *Aquifex aeolicus *VF5 and *Thermotoga maritima *MSB8^T ^is likely to impose similar constraints on amino acid composition and thus could cause an artificial clustering of these organisms. There are indeed indications that support an affiliation of *Aquifex aeolicus *VF5 with the *Proteobacteria *rather than with *Thermotoga maritima *MSB8^T ^[45]. Likewise, the association of the *Actinobacteria *and *Cyanobacteria *might be influenced by a biased amino acid composition as well [21].

A more in-depth discussion of the tree topology is beyond the scope of this paper. A much more detailed version of the ProML tree, showing all 120 species, can be obtained from the RibAlign website [39].

### Applicability

The applicability of phylogenetic reconstructions based on concatenated ribosomal proteins sequences has been discussed elsewhere in detail [20]. As with all protein-based phylogenies, concatenation of protein sequences has to face the problems of LGT and paralogy. LGT has been reported for some of the ribosomal protein encoding genes [46,47] and others do not qualify as makers because they have paralogs or are not universally present in all eubacteria. In addition, individual proteins in a concatenated alignment might evolve at different speeds, which requires the applications of more sophisticated likelihood-based models to account for this type of sequence heterogeneity [48]. Finally, site selection can have an impact on the positions of weakly supported branches of the inferred trees [20,25].

To be fair, most of these problems apply to the 16S rRNA approach as well. LGT of 16S rRNA genes is possible [49] and has been reported [50,51]. In addition, most bacteria have paralogs of the 16S rRNA gene that can differ considerably [52]. Also site selection has a major impact on the tree topology of 16S rRNA-based trees as well [6].

In the end, all trees that have been published so far based on concatenated ribosomal protein sequences are remarkably similar and mostly agree with the currently accepted 16S rRNA-based tree topology.

## Conclusion

Since the genomic revolution started in 1995 with the complete sequencing of *Haemophilus influenzae *Rd KW20 [53], new genomes are being sequenced at an exponentially increasing rate. This enables for new approaches in bacterial phylogeny that try to exploit a larger proportion from the genomic information for tree reconstruction than just single marker genes. To use such methods in an effective manner, a specialized and curated database of all potential marker genes from all genomes would be desirable.

RibAlign is a step in this direction for eubacterial ribosomal protein subunit sequences. We hope that it will be a helpful tool for scientists involved in whole genome sequencing of *Eubacteria*, particularly with regard to the phylogeny of representatives of new or only sparsely studied phyla.

## Availability and requirements

• Project name: RibAlign

• Project home page: 

• Operating system(s): Mac OS X

• Programming language: REALbasic front end on top of a Valentina object-relational database

• Other requirements: none

• License: license-free

• Any restrictions to use by non-academics: RibAlign may not be sold or bundled with any type of commercial application

## List of abbreviations

LGT – lateral gene transfer

megx – marine environmental genomics

MPI – message passing interface

PHYLIP – phylogeny inference package

RDP – ribosomal database project

rRNA – ribosomal ribonucleotide acid

## Authors' contributions

RibAlign was implemented by HT. FOG contributed important ideas regarding features, implementation, tested the program and was involved in the writing of the manuscript.

## Note

^1^MrBayes v 3.0B4 was used – version 3.1, which came out after our analysis, has lower memory requirements

^2^PHYLIP v. 3.6a4 was used
